# Heterogeneous GM-CSF signaling in macrophages is associated with control of *Mycobacterium tuberculosis*

**DOI:** 10.1038/s41467-019-10065-8

**Published:** 2019-05-27

**Authors:** Bryan D. Bryson, Tracy R. Rosebrock, Fikadu G. Tafesse, Christopher Y. Itoh, Armel Nibasumba, Gregory H. Babunovic, Bjorn Corleis, Constance Martin, Caroline Keegan, Priscila Andrade, Susan Realegeno, Douglas Kwon, Robert L. Modlin, Sarah M. Fortune

**Affiliations:** 1 Harvard T. H. Chan School of Public Health, 655 Huntington Avenue Boston, Boston, MA 02115 USA; 2 Ragon Institute of MGH, MIT, and Harvard, 400 Technology Square Cambridge, Cambridge, MA 02139 USA; 3 Oregon Health and Science University, 3181 SW Sam Jackson Park Rd, Portland, OR 97239 USA; 4 Division of Dermatology, David Geffen School of Medicine at University of California, California, USA

**Keywords:** Tuberculosis, Pathogens

## Abstract

Variability in bacterial sterilization is a key feature of *Mycobacterium tuberculosis* (Mtb) disease. In a population of human macrophages, there are macrophages that restrict Mtb growth and those that do not. However, the sources of heterogeneity in macrophage state during Mtb infection are poorly understood. Here, we perform RNAseq on restrictive and permissive macrophages and reveal that the expression of genes involved in GM-CSF signaling discriminates between the two subpopulations. We demonstrate that blocking GM-CSF makes macrophages more permissive of Mtb growth while addition of GM-CSF increases bacterial control. In parallel, we find that the loss of bacterial control that occurs in HIV-Mtb coinfected macrophages correlates with reduced GM-CSF secretion. Treatment of coinfected cells with GM-CSF restores bacterial control. Thus, we leverage the natural variation in macrophage control of Mtb to identify a critical cytokine response for regulating Mtb survival and identify components of the antimicrobial response induced by GM-CSF.

## Abstract (web summary)

GM-CSF has been implicated *in Mycobacterium tuberculosis* (*Mtb*) host control. Here, the authors show that activation of GM-CSF discriminates *Mtb*-restrictive from -permissive macrophages while co-infection with HIV disrupts GM-CSF-mediated signaling, rendering macrophages susceptible to bacterial infection.

## Introduction

M*ycobacterium tuberculosis* (Mtb), the causative agent of tuberculosis (TB), is the leading global cause of death due to infectious disease. Mtb is estimated to infect nearly a third of the global population but only causes overt disease in a subset of individuals. The natural variability in Mtb infection outcomes reflects the inherent capacity of the human immune response to control Mtb infection but also the fact that this immune control is clearly imperfect. To date, we have a limited understanding of the mechanistic basis of successful control or why this control sometimes fails in otherwise immunocompetent people^1^.

Macrophages are central mediators of the immune response to Mtb infection. They are one of the primary cell types infected with Mtb, and integrate a variety of immune signals to coordinate the response to infection. The best described pathway by which human macrophages can be activated to limit Mtb growth is through the vitamin D-dependent induction of antimicrobial peptides, cathelicidin and β-defensin 2^2^. The canonical activator, IFN-γ, which is strongly antimicrobial in murine macrophages through induction of nitric oxide production, drives human macrophages to kill Mtb through the activation of this vitamin D-dependent antimicrobial pathway^3^. It is unclear whether this pathway is the exclusive mechanism of Mtb killing by human macrophages or whether alternative pathways are available to some or all macrophage populations.

Since the 1960s it has been recognized that macrophages can be activated in response to different stimuli to generate different functional states. In the canonical paradigm, macrophages are polarized via different cytokine combinations into M1 or M2a/b/c states. M1 cells are characterized, broadly speaking, by microbicidal activity and M2 cells by a suite of immunoregulatory functions^4^. In the setting of Mtb infection, previously published studies have demonstrated that M1 macrophages are more restrictive of bacterial growth than M2 macrophages. While macrophage state is often simplified as a terminal differentiation state, dictated by the stimulus provided, emerging data supports a more nuanced model in which there is plasticity in cellular states and functional differences in the responses of apparently homogeneous myeloid cells exposed to the same stimulus^5^.

We developed a single cell model of Mtb killing by primary human monocyte-derived macrophages (MDMs). In our system, macrophages are matured in human serum without exogenous cytokine stimulation resulting in macrophages whose surface marker expression is similar that that of macrophages generated using M-CSF^6^. Using a bacterial live-dead reporter strain to assess macrophage antimicrobial capacity at a single cell level, we find that these macrophages display significant cell-to-cell variability in antibacterial capacity. We leveraged this natural variation in antimicrobial function to define the features of the antimicrobial pathways engaged in macrophages that naturally kill the infecting bacteria. Through global transcriptional profiling and transcriptional pathway analysis of isolated macrophage subpopulations, we find that differential expression of genes implicated in the GM-CSF signaling pathway most strongly discriminates macrophages that have successfully controlled Mtb (restrictive macrophages) from macrophages that permit bacterial survival (permissive macrophages) and that addition of exogenous GM-CSF further increases bacterial killing. GM-CSF mediated bacterial killing correlates with increased phagolysosomal maturation but not increased CAMP expression or reactive nitrogen species production, suggesting that it activates a different antimicrobial pathway than that activated by vitamin D and IFN-γ. Finally, we show that human immunodeficiency virus (HIV) coinfection makes macrophages more permissive of Mtb growth, which correlates with reduced GM-CSF production, and that treatment with GM-CSF, but not other canonical activators such as vitamin D or IFN-γ, restores bacterial killing by HIV-TB coinfected cells. Taken together, these data define a pathway to bacterial control in human macrophages.

## Results

### Macrophage control of Mtb infection is heterogeneous

**Fig. 1:**
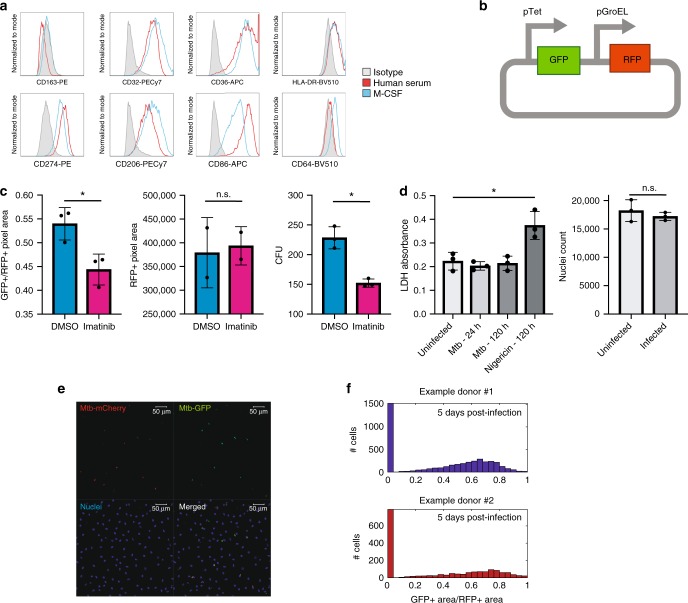
Primary monocyte-derived macrophages have variable capacity to restrict Mtb. **a** Representative flow cytometry plot of surface expression of CD163, CD32, CD36, HLA-DR, CD274, CD206, CD86, CD64 on human serum or M-CSF-derived macrophages **b** Plasmid design for transcriptional live-dead reporter. **c** Quantification of bacterial viability (GFP + area/RFP + area) in macrophages stimulated with DMSO or imatinib. Comparisons made using a two-tailed *t*-test. **d** Quantification of macrophage viability by LDH release and nuclei counting following Mtb infection. Comparisons made using a two-tailed *t*-test. **e** Example confocal image of macrophages infected with Mtb containing a strain that constitutively produces the fluorophore mCherry and produces GFP upon tetracycline induction. DAPI illuminates macrophage nuclei. **f** Example histograms of bacterial viability (GFP + area/RFP + area) measurements across individual cells in two donors. Representative of > 10 donors. (ns not significant, *, *p* < 0.05) Source data are provided as a Source Data file

In primary human monocyte-derived macrophages (MDMs) the total Mtb burden increases slowly over time, which has historically been interpreted as evidence that in the absence of sufficient activation of the vitamin D pathway, primary human MDMs slow Mtb growth but cannot kill the bacteria^3,7^. However, more recent studies suggest that the slow increase in Mtb burden measured at a population level instead represents the integrated effects of simultaneous bacterial growth and killing^8^. We differentiated CD14^+^ monocytes into MDMs using human serum in the absence of additional exogenously added cytokines that would strongly skew the population. We compared the surface expression of CD36, HLA-DR, CD163, CD32, CD86, CD274, and CD206 between human-serum differentiated macrophages and macrophages matured with M-CSF. We observed that compared to an isotype-control, both M-CSF and human-serum derived macrophages expressed all of these markers with the exception of CD163 although there were slightly different levels in surface expression of these markers (Fig. 1a).

We used human serum differentiated macrophages to directly assess macrophage antimicrobial function at a single cell level with the goal of disentangling Mtb growth in aggregate from single-cell dynamics of bacterial killing. To do this, we utilized an H37Rv reporter strain carrying a live-dead plasmid that we and others have previously shown enables measurement of bacterial fate with single cell resolution using high density microscopy (Fig. 1b, Supplementary Fig. 1)^9–11^. Briefly, in this strain a long-lived red fluorescent protein (E2-Crimson or mCherry) is constitutively expressed under the control of the GroEL promoter, marking the presence of all bacteria. To assess bacterial viability, green fluorescent protein (GFP) is expressed under the control of the tetracycline (Tet) inducible promoter. Bacteria that respond to inducer and express GFP are considered transcriptionally active, which we use as a proxy for viability (live bacteria). We validated the reporter as a metric for host mediated clearance by treating macrophages with imatinib, a well-described host directed therapy for tuberculosis^12,13^. Imatinib treatment led to a decline in viable bacteria as assessed by the reporter and consistent with the measured change in CFU (Fig. 1c). We also assessed the relative stability of the signal of the red fluorescent protein in our system over time to identify clearance of dead bacteria that might lead us to significantly underestimate bacterial killing with the reporter system as certain antibiotics can rapidly diminish all fluorescent protein signal by disrupting the bacterial cell wall. Tracking the red signal over time revealed that there was no precipitous loss of RFP signal on a per cell basis over the first five days after imatinib treatment. Finally, we defined infection conditions under which Mtb infection did not lead to significant death as loss of cells would impact our ability to interpret bacterial survival dynamics. We used two strategies to measure macrophage viability, following both LDH release over time and the total number of nuclei per well. When macrophages are infected at a multiplicity of infection of one, we did not detect a reduction in the total number of nuclei per well or an increase in the release of LDH although we were able to detect enhanced LDH release using nigericin, a potent activator of pyroptosis that is known to drive LDH release (Fig. 1d).

Using this system, we tracked Mtb survival in macrophages over time at a single cell level. Prior to infection and at early time points post infection, the population of Mtb bacteria is almost entirely viable. However, over time an increasing fraction of the macrophages appear to have fully killed the infecting bacteria while others continue to harbor fully viable Mtb (Fig. 1e, f). In addition, some macrophages contain a mixture of transcriptionally active and silent bacteria, perhaps reflecting the temporal dynamics inherent in the system.

### RNAseq reveals distinct macrophage programs during infection

**Fig. 2:**
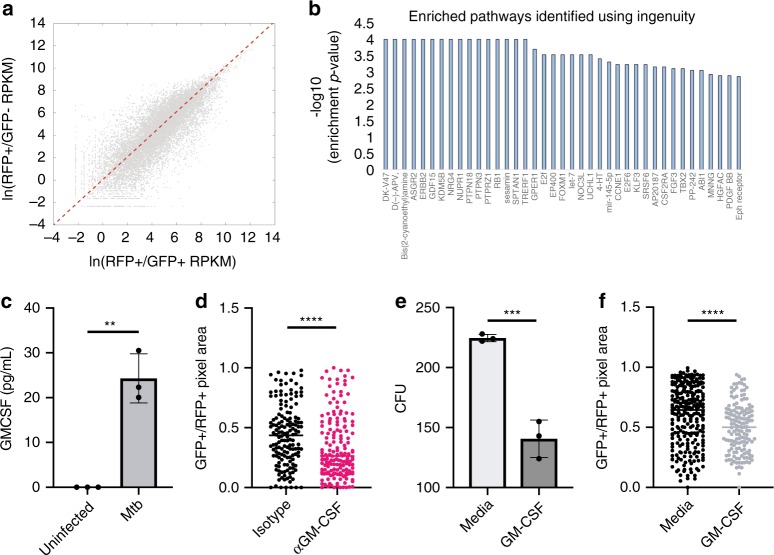
Transcriptional profiling reveals GM-CSF as critical regulator of macrophage control of Mtb. **a** Scatter plots comparing gene expression between RFP + /GFP- macrophages to RFP^+^/GFP^+^ macrophages from the same culture. **b** Bar graph summarizing results of pathway analysis of differentially expressed genes in **a** using Ingenuity Pathway Analysis identifying the CSF2RA (GM-CSF signaling) pathway as enriched in differentially expressed genes. **c** Extracellular GM-CSF was measured and compared between paired Mtb-only and uninfected cultures at 24 h post-Mtb infection. Error bars represent standard deviation of the technical replicates. Comparisons made using a two-tailed t-test. Representative of three donors. **d** Mtb-infected macrophages were scored 4 days post-infection based on the percentage of transcriptional activity following pre-treatment with anti-GM-CSF antibody or isotype control. Dots represent data from an individual macrophage. Black lines represent the median. Comparisons made using the Mann–Whitney test. Representative of three donors. **e** Measurements of bacterial survival after GM-CSF treatment using colony-forming units. Error bars represent standard error from three technical replicates. Representative of six donors. Comparisons made using a two-tailed t-test. **f** Mtb-infected macrophages were scored 4 days post-infection based on the percentage of transcriptional activity of the untreated or GM-CSF treated macrophages. Black lines represent the median. Dots represent data from an individual macrophage. Comparisons made using the Mann–Whitney test. Representative of six donors. (**p < 0.01, ***p < 0.001, ****p < 0.0001). Source data are provided as a Source Data file

We sought to identify molecular differences underlying this heterogeneity in macrophage functional capacity, isolating functionally distinct macrophage populations by flow cytometry where we sorted based on the state of the bacterial transcriptional reporter. Briefly, macrophages were infected with the reporter Mtb strain MOI of 0.5, and then infection was allowed to proceed for 4 days. Anhydrotetracycline was added at day 4 to induce the live-dead reporter prior to sorting. Uninfected macrophages were used to establish gates for RFP^−^/GFP^−^. Three populations of macrophages were isolated: (1) RFP^-^/GFP^-^, (2) RFP^*+*^/GFP^*-*^ (restrictive) and (3) RFP^*+*^/GFP^*+*^ (permissive). We analyzed at least 5000 cells per condition from three independent donors using low-input RNA sequencing to identify genes and pathways that were differentially expressed or active between these distinct subpopulations. As expected, we observed transcriptional differences between the sorted subpopulations (Fig. 2a, Supplementary Data 1). In both restrictive and permissive infected cells, we observed regulation of metabolic genes such as *CYP20A1*, components of the interferon signaling pathway *IFIT1* and *IFIT3*, and transcriptional regulators such as *IRF7*, a critical regulator of the immune response downstream of TLR signaling^14^ (Supplementary Data 1). We also identified a suite of differentially expressed genes that distinguished the restrictive and permissive populations of macrophages including *NLRP3*, *IL4R*, *CLEC4A*, and *FCGR3A* which were differentially expressed across multiple donors (Supplementary Data 1). Taken together, these differentially expressed genes point to differences in the inflammatory state of these macrophage subpopulations.

### GM-CSF enhances control of Mtb

To gain insight into the signaling pathways underlying the emergence of these distinct subpopulations, we utilized pathway analysis to identify pathways preferentially activated in restrictive macrophages instead of leveraging a gene-centric approach to deciphering candidate pathways that distinguish restrictive and permissive macrophages. Using Ingenuity Pathway Analysis, we sought to identify candidate pathways that were enriched in the differentially expressed genes. We analyzed those genes whose average expression changed by two-fold or more (up or down) in the three donors analyzed. Several candidate pathways were identified that significantly distinguished restrictive and permissive cells including *NUPR1*, *FOXM1*, and *CSF2RA*, also known as the GM-CSF receptor (Fig. 2b, Supplementary Data 2).

We focused on the effects of GM-CSF signaling given the availability of biological tools available to probe this signaling axis in human macrophages. GM-CSF has long been known as a critical hematopoietic growth factor; however, recent studies have identified a role for GM-CSF in homeostatic and inflammatory conditions consistent with our transcriptional analysis^15–17^. In our system, macrophages are matured via adherence in the absence of exogenously added cytokines. To assess the function of GM-CSF in the setting of Mtb infection, we first measured the secretion of GM-CSF in response to Mtb infection. Cells infected with Mtb but not uninfected macrophages secrete low but measurable quantities of GM-CSF as measured by ELISA by 24 h post-infection (Fig. 2c, Supplementary Fig. 2a). Neutralization of endogenous GM-CSF reduces macrophage killing of Mtb as compared to treatment with an isotype control antibody (Fig. 2d, Supplementary Fig. 2b). Moreover, addition of exogenous GM-CSF increased the capacity of the macrophage population to kill Mtb as demonstrated by the live-dead reporter assay and by measuring colony-forming units (CFU) (Fig. 2e, f, Supplementary Fig. 2c, Supplementary Fig. 2d). These data demonstrate that human macrophages produce GM-CSF in response to infection, which enhances the antimicrobial activity of at least some macrophages in the population. We note that GM-CSF did not enhance Mtb control in every macrophage suggesting that the quality of the GM-CSF response within the macrophage population was not equivalent between all cells, although we could not find evidence for differences in the expression of the GM-CSF receptor of GM-CSF itself in restrictive and permissive cells to explain these differences (Supplementary Data 1).

### GM-CSF enhances antimicrobial programs

**Fig. 3:**
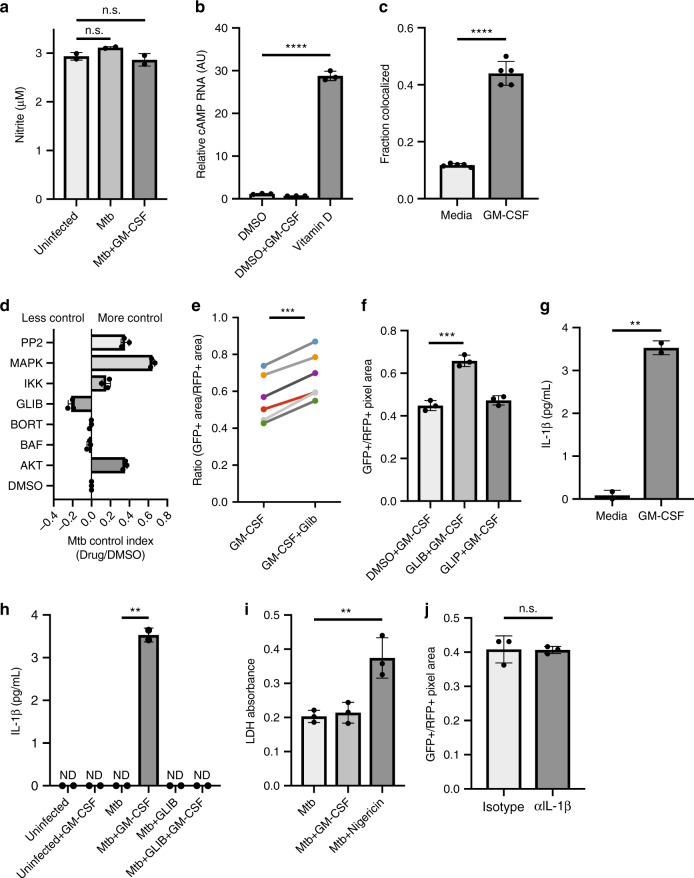
GM-CSF stimulates phagolysosomal fusion and IL-1β secretion in the absence of antimicrobial peptide production or NO production. **a** Nitrite production by uninfected, Mtb-infected, and GM-CSF stimulated Mtb-infected macrophages. Representative of three donors. **b** Quantitative PCR analysis of *CAMP* mRNA expression normalized to GAPDH expression in primary human macrophages were treated with GM-CSF or active Vitamin D. Each value represents the mean of ± standard deviation of three independent experiments. **c** Phagolysomal colocalization of GFP-Mtb with Lysotracker red was measured in 200–600 macrophages per well. Representative of three donors. **d** Small molecule GM-CSF inhibitor screen example. Bars pointing to the right identify drugs that had an increase in bacterial control while bars pointing to the left indicate a decrease in the setting of GM-CSF. **e** The effect of glibenclamide on GM-CSF mediated control was compared for six independent experimental donors (each donor independently color-coded). **f** The effect of glibenclamide or glipizide on GM-CSF mediated control was compared between vehicle-, glipizide- or glibenclamide-treated cultures. Representative of three donors. **g** Extracellular IL-1β production was measured from unstimulated and GM-CSF stimulated Mtb-infected macrophages. Error bars represent standard deviation of the technical replicates. Representative of three donors. **h** Extracellular IL-1β production was measured from unstimulated and GM-CSF stimulated Mtb-infected macrophages with small molecule inhibitors. Error bars represent standard deviation of the technical replicates. Representative of three donors. **i** LDH absorbance values in Mtb-infected macrophages with vehicle, GM-CSF or nigericin. Error bars represent standard deviation of the technical replicates. Representative of three donors. **j** Quantification of bacterial survival in the setting of GM-CSF with isotype or IL-1β antibody. Error bars represent standard deviation of the technical replicates. Representative of three donors. All comparisons made using a two-tailed *t*-test. (ns, not significant, **p < 0.01, ***p < 0.001, ****p < 0.0001). Source data are provided as a Source Data file

We next sought to define the pathways underlying GM-CSF-driven Mtb control in MDMs. We began by testing well-established mechanisms that mediate bacterial control, production of reactive nitrogen species and production of antimicrobial peptides. Nitric oxide is a key antimicrobial effector and well-recognized feature of classically defined M1 murine macrophages; however, production of reactive nitrogen species by human macrophages has been difficult to detect, and recent data suggests that observed increases in reactive nitrogen species are of bacterial origin^18,19^. Nevertheless, given the literature describing nitric oxide as an antimicrobial mediator, we sought to assess the production of nitric oxide in our system. In the bulk using a Griess assay, we found no detectable increase in production of nitrite with GM-CSF consistent with an NO-independent mechanism (Fig. 3a). We were also unable to detect NOS_2_ by immunofluorescence or any evidence of NOS_2_ expression in our sorted populations of macrophages with or without GM-CSF treatment (Supplementary Fig. 3). Alternative pathways implicated in human macrophage control of Mtb include the production of antimicrobial peptides in the setting of IFN-γ and Vitamin D exposure^2^. We tested whether GM-CSF induced the expression of cathelicidin during Mtb infection as a mechanism for Mtb control using quantitative RT-PCR. Consistent with published literature, macrophage stimulation with active vitamin D induced a pronounced increase in cathelicidin transcript at 24 h (Fig. 3b, Supplementary Fig. 4a)^20^. However, we saw no increase in cathelicidin upon GM-CSF treatment suggesting cathelicidin was not the mediator of GM-CSF induced Mtb control although we cannot fully exclude regulation of cathelicidin levels in a subpopulation of cells.

Phagolysosomal fusion has also been implicated as a mechanism for enhanced Mtb control, and several Mtb effector proteins have been described to disrupt phagolysosomal fusion as well as lysosomal acidification^21,22^. To assess the effects of GM-CSF on phagolysosomal fusion, we stained unstimulated or GM-CSF stimulated Mtb-infected macrophages with the pH-sensitive dye Lysotracker red and imaged these cells using confocal microscopy. GM-CSF stimulation enhanced Mtb localization in acidic compartments consistent with previous observations in mice suggesting neutralization of GM-CSF in vivo diminishes phagolysosomal fusion and enhances bacterial survival (Fig. 3c, Supplementary Fig. 4b). This suggests that GM-CSF may act to either enhance trafficking of Mtb to the lysosome or enhancing an alternative antimicrobial response, which then subsequently results in trafficking of Mtb to the phagolysosome.

In parallel with these directed strategies to explore the relationship between GM-CSF stimulation and known antimicrobial programs, we attempted to an agnostically understand the mechanisms of GM-CSF mediated control. We were unable to take a genetic approach because, in our hands, human macrophage cell lines are not capable of robust Mtb killing and nucleofection of MDMs followed by Mtb infection lead to extensive cell death. Therefore, we used a panel of small molecule inhibitors to target specific aspects of the response to infection in MDMs. Macrophages were infected with the live-dead Mtb reporter strain, treated with inhibitor and then treated with exogenous GM-CSF. Bacterial survival was quantitated four days post infection by high-density microscopy. Because our endpoint was bacterial survival, we focused on small molecules that did not cause significant macrophage death over the course of the assay.

We calculated a killing index by calculating the bacterial viability measured after treatment with the small molecules and the bacterial viability after treatment with a vehicle control. The killing index is a ratio of the viability in the small molecule treated condition relative to the vehicle control. Most of the small molecules had either no effect on or enhanced bacterial killing by our live-dead reporter. However, glibenclamide, an NLRP3 inhibitor attenuated the ability of GM-CSF to activate human macrophages to kill Mtb consistently across multiple donors (Figs. 3d, e)^23,24^. To control for off-target effects of glibenclamide, we also tested the effect of glipizide, a structurally similar drug which does not inhibit NLRP3, and found that it had no effect on GM-CSF driven bacterial control (Fig. 3f, Supplementary Fig. 4c)^23^. NLRP3 inhibition has been associated with diminished IL-1β secretion, so we next sought to measure IL-1β in the setting of glibenclamide and GM-CSF. Upon GM-CSF stimulation, we found a modest increase in IL-1β secretion, and consistent with a model for NLRP3-mediated IL-1β secretion, we found that glibenclamide treatment reversed IL-1β secretion (Fig. 3g, h, Supplementary Fig. 4d). IL-1β secretion is often associated with LDH release and pyroptosis; however, in our system, GM-CSF treatment activates bacterial killing as measured at a single cell level via microscopy in intact macrophages, suggesting that pyroptosis was not artificially leading to a decrease in Mtb numbers. Consistent with this observation, measurement of LDH release as an indicator of cell death did not reveal differences between LDH release by Mtb infected macrophages treated with GM-CSF and untreated macrophages despite differences in IL-1β secretion; however, increased LDH release was seen with nigericin, an alternative driver of IL-1β secretion (Fig. 3i, Supplementary Fig. 4e). Neutralization of IL-1β had no impact on bacterial killing suggesting that IL-1β may serve as an environmental inflammatory cue as opposed to a mechanism to enhance cell-intrinsic bacterial control (Fig. 3j, Supplementary Fig. 4f).

### HIV co-infection disrupts GM-CSF secretion and Mtb control

**Fig. 4:**
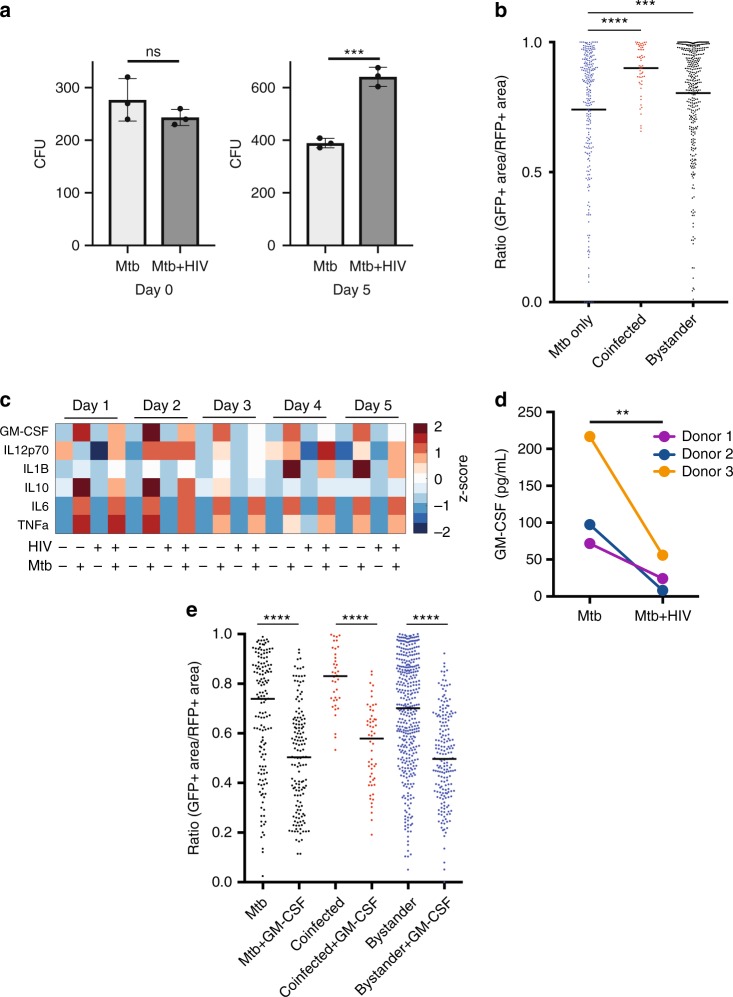
HIV coinfection disrupts macrophage control of Mtb and disrupts GM-CSF signaling. **a** One of two paired human MDM cultures was coinfected (infected with HIV and subsequently Mtb (Mtb + HIV)), while the other was infected with Mtb only. Uptake and growth of Mtb was compared using colony-forming units (CFU) at Day 1 and Day 5 post-infection, respectively. Error bars represent standard error from technical replicates. Comparisons made using a two-tailed *t*-test. Representative donor of four. **b** Mtb-infected macrophages were scored 5 days post-infection based on the percentage of transcriptional activity of the resident Mtb at various times post-infection. Dots represent data from an individual macrophage. Black lines represent the median. Comparisons made using the Mann-Whitney test. Representative of six donors. **c** Multiplex analysis of GM-CSF, IL-12, IL-1β, IL-10, IL-6, and TNFα secretion was measured and compared between uninfected, HIV only, Mtb-only and co-infected cultures at various times post-Mtb infection. **d** The secretion of GM-CSF 1 day post-infection was compared between paired Mtb-only and coinfected cultures. Three independent experimental donors are represented. Comparisons made using a paired two-tailed *t*-test. **e** Mtb-infected macrophages were scored 4 days post-infection based on the percentage of transcriptional activity of the resident Mtb and compared to paired co-infected cells, with or without treatment with GM-CSF. Dots represent data from an individual macrophage. Black lines represent the median. Representative donor of six. Comparisons made using the Mann–Whitney test. (ns = not significant, **p < 0.01, ***p < 0.001, ****p < 0.0001). Source data are provided as a Source Data file

We focused on the pathways underlying natural variation in macrophages’ antimicrobial capacity because we postulated that these might be highly vulnerable to modulation. In parallel studies, we sought understand the effects of HIV on macrophage control of Mtb infection and surprisingly these studies also converged on the importance of GM-CSF in driving macrophage control of Mtb. Like Mtb, HIV has the capacity to infect macrophages and we and others have postulated that the potential interactions between Mtb and HIV may contribute to the accelerated TB disease observed in HIV-infected individuals^25,26^. To test this model, we developed an in vitro model of coinfection using human MDMs infected with the virulent HIV strain HIV-bal. Our single cell reporter assay is of particular value in this experimental setting where HIV and Mtb both infect subpopulations of macrophages with considerable donor-to-donor variation. A single-cell assay where we can specifically assess Mtb-infected or Mtb/HIV-coinfected cells allows us to detect more subtle differences where the bulk of the population of cells may not be infected with either the bacterium or virus. HIV was permitted to replicate over two weeks after which cells were infected with Mtb. Infection with HIV resulted in a notable increase in surface expression of CD36 and CD32 however there were not consistent changes in the expression of other surface markers across all donors when comparing sham to HIV infected cells (Supplementary Fig. 5). Macrophage uptake of bacteria was not influenced by HIV infection (Fig. 4a). However, significantly higher Mtb burden was observed on day 5 in macrophage cultures infected with HIV as compared to HIV uninfected cells (Fig. 4a). We assessed the effect of HIV infection on macrophage control of the bacteria at a single cell level using the live-dead Mtb reporter strain and p24 immunofluorescence to identify macrophages supporting HIV replication. Macrophages that were positive for p24 and Mtb (coinfected macrophages) and macrophages that were negative for p24 and positive for Mtb (bystander macrophages) were both significantly more permissive of Mtb survival than macrophages not exposed to HIV (Fig. 4b).

Because HIV altered macrophage control of Mtb in bystander cells as well as in co-infected cells, we postulated that HIV alters the macrophage cytokine response to Mtb infection. Therefore, we quantified the production of cytokines produced by macrophages (IL-6, IL-10, TNFα, IFN-γ, GM-CSF and IL-1β□ in non-infected, Mtb-infection, HIV-infected and co-infected cultures over the course of an Mtb-infection using multiplex cytokine profiling (Fig. 4c). Strikingly, of the cytokines measured, only GM-CSF, IL-1β and IL-10 were differentially secreted in co-infected cultures compared to Mtb cultures not exposed to HIV. Interestingly, while the difference in IL-10 secretion was only detected at early time points and the difference in IL-1β secretion was only detected at later time points, GM-CSF production was significantly lower in co-infected macrophage cultures at early as well as later time points. We confirmed the GM-CSF secretion result by ELISA in three additional experimental donors (Fig. 4d).

We next sought to test our model that GM-CSF enhances Mtb control in HIV coinfected macrophages. We treated cultures of coinfected macrophages with GM-CSF or IL-1β. Treatment with GM-CSF but not IL-1β led to significantly better Mtb killing in HIV infected macrophage cultures (Fig. 4e, Supplementary Figs 6 and 7). Importantly, only GM-CSF treatment and not treatment with other canonical macrophage activators (Vitamin D, TNFα, IFN-γ) activated HIV infected macrophages to kill Mtb (Supplementary Figs 6 and 7). Previous reports have demonstrated that GM-CSF can reduce macrophage permissivity to HIV, so we sought to test the hypothesis that enhanced Mtb control was driven by reduced HIV burden. Consistent with previous literature, we found that GM-CSF treated macrophages had reduced p24 staining (Supplementary Fig. 8a)^27^. Active vitamin D (1,25 dihydroxy vitamin D) treated cells, also had reduced p24 staining; however, Vitamin D treated cells were unable to enhance Mtb control in coinfected macrophages (Supplementary Fig. 8b). Taken together, these data suggest a unique and pivotal role for GM-CSF in the control of Mtb infection.

## Discussion

Macrophages represent a functionally diverse cell type whose encounters with pathogen can ultimately shape the course of infection. Our studies reveal that primary human macrophages vary in their capacity to kill Mtb. We sought to leverage the natural variation in human macrophage control of Mtb as a tool to dissect the dominant pathways that contribute to bacterial killing. While genetic screens have proven to be powerful techniques to dissect complex systems when coupled with a selectable marker, genetic screens in primary human macrophages are complicated by the technical challenges of targeting these cells and the fact that genetic manipulation can activate innate defenses. Instead, we sought to exploit the natural variation in our system to identify natural dominant drivers of outcome. Activation of the GM-CSF signaling pathway characterized macrophages that were successful at controlling bacterial fate.

Our findings extend previous work suggesting a role for GM-CSF in the control of Mtb infection. In murine macrophages, GM-CSF has been implicated in cell-intrinsic control of mycobacterial infection^28^. Mice deficient in GM-CSF succumb quickly to tuberculosis infection^29^. However, these mice, like humans with abnormalities in the GM-CSF receptor, suffer from pulmonary alveolar proteinosis due to defects in alveolar macrophage clearance of surfactant. This creates structural lung disease that masks a possible immunologic contributions to infection risk, although patients with acquired immunodeficiency caused by the production of autoantibodies to GM-CSF^30,31^ appear to be at very high risk of nontuberculous mycobacterial infection and a moderately increased risk of TB even in the absence of overt pulmonary alveolar proteinosis^27,28^. Additionally, GM-CSF has been revealed to enhance control of additional pathogens such as *Candida albicans* suggesting that GM-CSF may be able to enhance control of multiple intracellular pathogens^32^. Furthermore, another group has reported a unique role for GM-CSF in enhancing the control of Mtb in human macrophages by optimizing macrophage differentiation and culture conditions^33^. Our data, however, are in contrast to recent reports that suggest no difference in Mtb growth in human macrophages differentiated using M-CSF or GM-CSF. It is possible that prolonged tonic differentiation in M-CSF or GM-CSF induce a tolerized macrophage state as opposed to a macrophage that sees a high-dose of GM-CSF following infection^34,35^.

Our data suggest that an important immunologic role of GM-CSF may be to modulate the inflammatory response through activation of antimicrobial pathways as well as through amplification of an inflammatory environment via IL-1β secretion. While the murine pathways implicated in GM-CSF mediated control of Mtb involve PPARγ and nitric oxide, our data do not identify a role for nitric oxide in the setting of Mtb control, highlighting known differences between murine and human antimicrobial pathways^36,37^. Despite this discrepancy, our data in consistent with the described role for GM-CSF in promoting phagolysosomal fusion in murine macrophages.

Our data point to a unique and important role for GM-CSF in control of Mtb infection. In the absence of HIV infection, we demonstrate that GM-CSF is produced by human macrophage within hours of Mtb infection and that exogenous GM-CSF activates human macrophages to kill Mtb. HIV infection specifically inhibits macrophage production of GM-CSF but not the ability of the macrophage to respond to GM-CSF repletion. By contrast, HIV infection blocks the ability of the Mtb infected macrophage to kill Mtb in response to Vitamin D_3_, IL-1β, IFN-γ and TNF-α both alone and in combination^38^.

A previously published study suggested that Vitamin D treatment of macrophages at the time of simultaneous HIV and Mtb infection limits both HIV and Mtb infection^39^. The discrepancy in our findings may reflect differences in timing of intervention and infection as we designed our assay system to reflect a situation in which infection is established before treatment is initiated. Indeed, our data may provide some insight into the failure of Vitamin D supplementation to consistently improve clinical outcomes in TB and HIV infected individuals in clinical studies.

This work demonstrates that GM-CSF signaling is an important mechanism to tune macrophage inflammatory state and improve mycobacterial control and that this pathway can be manipulated by pathogens to drive a more permissive bacterial state.

## Methods

### *M. tuberculosis* and HIV cultures

The *M. tuberculosis* H37RV transcriptional reporter strain was grown in Difco Middlebrook 7H9 media supplemented with 10% OADC, 0.2% glycerol, 0.05% Tween-80 and Hygromycin B (50 µg/ml) to an OD_600nm_ (OD) of 1. The H37Rv was a gift from Barry Bloom. Two days before macrophage infection, the Mtb culture was diluted to an OD of 0.01 to reach a final OD of 0.4. HIV stocks were prepared from the supernatant of a U87 cell line culture infected with virulent HIV-BaL in DMEM media + 10% FCS. U87 cells were obtained from ATCC, and HIV-bal was a gift from Anne Goldfeld. HIV stocks were stored at −80 °C. HIV titers were measured using the U87 cell line infected with various serial dilutions of the harvested HIV supernatants and the formation of syncytia after 7 days of incubation was scored.

### Monocyte-derived macrophage isolation

Human monocytes were isolated from human buffy coats purchased from Research Blood Components (Watertown, MA) using a standard Ficoll gradient and subsequent CD14 + cell positive selection (Stemcell Technologies, Tukwila, WA). Selected monocytes were cultured in low-adherence flasks (Corning) for 5 days with RPMI media (Invitrogen) supplemented with 10% human serum (Valley Biomedical, Winchester, VA) prior to infection with HIV or Mtb.

### Mtb infection and HIV coinfection

For Mtb infections not involving HIV coinfection, monocyte-derived macrophages (MDM) were infected with Mtb at an MOI of 1:1 (Mtb:cells) for 4 h in RPMI + 10% heat-inactivated FCS (FCS). After 4 h, cells were washed twice with media and then cultured in the appropriate cell culture media with drugs or cytokines added as necessary. Infection was allowed to proceed for 4 days prior to the addition of anhydrotetracycline (100 ng/mL) for 24 h prior to fixation with 4% paraformaldehyde for 2 h at room temperature.

For HIV coinfections, half of the monocyte-derived macrophages (MDM) isolated per donor were infected with HIV four days post-isolation at a MOI of 1:10 (cells:virions). Briefly, all MDM cells were concentrated in 500ul of RMPI media (Invitrogen) + 10% human serum (Valley Biomedical, Winchester, VA) with polybrene (4ug/ml). Half of the cells were incubated with the virus for 2–3 h, and then both infected and uninfected macrophages were washed twice with 50 mL of RPMI. Finally, the two cell cultures were incubated separately in low-adherence 6-well culture dishes in RPMI + 10% human sera for 21 days, with media refreshment every 4 days. Infection with Mtb occurred 21 days post-HIV infection. Briefly, the Mtb culture was washed twice with RPMI + 10% FCS and filtered through a 5um syringe filter. MDM were infected at an MOI of (1:1) and washed twice with RPMI + 10% FCS four hours post-infection and infection was allowed to proceed for various amounts of time depending on experimental design.

### Intracellular staining

To visualize cell nuclei and intracellular HIV protein, paraformaldehyde-fixed MDM cells were treated with 0.1% Igepal and 1% saponin for 20 min, washed with 1× PBS and incubated for 1 hour with human sera as a blocking agent. Cells were then incubated with (500 ng/ml) of DAPI in 0.1% Igepal for 10 min and washed twice with 1× PBS. Anti-p24 monoclonal antibody (Thermo Scientific, MA1–7040) was conjugated to Alexa-Fluor 647 (Invitrogen) as per manufacturer’s instructions and purified by column chromatography (GE Healthcare) followed by desalting columns (Thermo Scientific). MDM were stained with 0.1 μg/ml monoclonal antibody for 1 hour, washed with 1× PBS three times and coated with 50% glycerol + n-propyl gallate (5 mg/ml) for imaging.

To visualize iNOS expression in Mtb-infected macrophages, macrophages were fixed for 2 h with 2% PFA at room temperature (RT). After fixation, the samples were blocked in 0.3% Triton-X 100/5 % donkey serum serum/1X PBS for 60 min at RT, washed, and incubated with anti-NOS2 (Cell Signaling Technology Clone D6B6; 1:100) overnight at 4 °C. The samples were washed again and stained with an Alexa Fluor 647–conjugated donkey anti-rabbit secondary antibody (Thermo Fisher; 1:1000, A-31573) for 1 h at RT. Images were obtained using a Zeiss LSM 510 Confocal Microscope fitted with a 20X objective.

### Imaging and analysis

MDM cells and the resident HIV and Mtb were imaged using a DeltaVision fluorescence microscope (GE Healthcare) with Z-stack of 0.5 slice thickness over 15 µm. Individual cells were demarked using a brightfield image taken at the midpoint of the Z-stack. Fluorescence Z-stack images were averaged for each light channel. To accurately detect and measure Mtb signal, cell background per macrophage was measured in ImageJ for both the 580 (mCherry) and 525 (GFP) wavelengths. Pixel intensities 4 standard deviations above the average cell background signal in the 580 channel was considered positive signal for Mtb mCherry (constitutive signal), while pixel intensities with values above 2 standard deviations the average cell background in the 525 channel were scored as positive for Mtb GFP signal (transcriptional induction). Only pixels positive for Mtb mCherry signal were measured for GFP signal. The transcriptional activity of Mtb per macrophage was quantified by dividing the total intracellular pixels scored positive for mCherry signal by the total number of pixels scored positive for GFP signal in the same macrophage. Fluorescence intensity in the 647 channel was also measured for individual macrophages in cultures infected and not infected with HIV. Intensity value per cell was divided by total cell area. A macrophage was scored positive for active HIV replication when 647 signal was greater than 2.5 standard deviations above the average signal for macrophages not exposed to HIV.

Small molecule inhibitor effects on bacterial viability were analyzed by high-throughput high-density microscopy. The cells were washed, fixed, and stained with DAPI. Images were obtained via Operetta High-Content Imaging Fluorescence Microscope (Perkin-Elmer) outfitted with 20 × NA objective. Total Mtb bacterial burden was determined based on mCherry + pixels. Transcriptionally active Mtb bacterial burden was determined based on GFP + pixels. Between 1.5 × 10^4^ and 4 × 10^4^ cells per condition over technical triplicates per donor were analyzed using Columbus (Perkin-Elmer). Bacterial survival was calculated as a ratio of live to total bacteria (the number of GFP + pixels (live) divided by the number of mCherry + pixels (total burden). Experimental conditions where the number of nuclei decreased dramatically relative to a vehicle control were excluded from subsequent analyses.

For co-localization of Mtb in lysosomes, GM-CSF (5 ng/mL) was added to MDM infected with GFP-expressing Mtb as described above for 24 hr at 37 °C. Lysotracker Red DND-99 (1 mM; Thermo Fisher) was added to samples for 1 hr at 37 °C prior to washing and fixation. Macrophage nuclei were stained with DAPI (Sigma; 500 ng/mL) in 0.1% IGEPAL CA-630 (Sigma) for 10 min at RT, and the cells were washed. Images were obtained using a Zeiss LSM 510 Confocal Microscope fitted with a 20× objective. Between 200 and 600 macrophages per condition over technical duplicates per donor (total three) were analyzed using ImageJ.

### Colony forming units

Mtb-infected macrophages were lysed with 1× PBS + 0.05% Triton x-100 for 5 min to release intracellular Mtb. The lysis supernatant was diluted 10 fold with Difco Middlebrook 7H9 media and stored at −80 °C until plating on Difco Middlebrook 7H10 media + Hygromycin B (50 µg/ml) to determine Mtb growth. 7H10 plates were incubated at 37 °C for 3 weeks.

### Cytokine, small molecule and antibody treatment

All macrophage cytokine or activator treatments occurred 1 day post Mtb infection. GM-CSF (R&D systems) was added at 5 ng/ml, IL-1β (Biolegend) was added at 10 ng/ml, active Vitamin D (1,25 dihydroxy Vitamin D) (Enzo) was added at 10^–7^ M, IFN-γ (Sigma) was added at 10 ng/ml, and TNF-α (R&D systems) was added at 100 ng/ml. Leaf-purified anti-human GM-CSF antibody (Biolegend, 502203) was added at a concentration of 10 μg/ml, as was the IgG2a isotype control antibody (Biolegend, 400515), ~10 min before Mtb infection. Anti human IL-1β antibody (R&D systems, MAB601) was added at a concentration of 10 μg/mL, as was the IgG1 isotype control (R&D systems, MAB602). All small molecules with the exception of bafilomycin, nigericin, and glibenclamide were use at 10 μM final concentration. Glibenclamide was used at a final concentration 50 μg/mL, nigericin was used at a final concentration of 5 μM and bafilomycin was used at a final concentration of 1 μM.

### Cytokine measurements

Seven cytokines were measured in macrophage cell culture supernatants using the Human inflammation cytokine/chemokine magnetic Milliplex MAP kit (Millipore Corporation). The following analytes were measured: interleukin-1 beta (IL-1β), IL-4, IL-6, Il-8, IL-10, IL-12p70, interferon-γ (IFNγ), tumor necrosis factor α (TNFα) and granulocyte macrophage colony-stimulating factor (GM-CSF) according to the manufacturer’s instructions. The sensitivity of the kit ranged from 0.0005 to 0.2 ng/ml depending on the analyte measured. Undiluted cell culture supernatant samples were harvested from macrophage cultures at various times in the experiment and stored at −80 °C. Supernatants were thawed and filtered twice, to remove Mtb, by centrifugation using 0.2 µm Bio-Inert AcroPrep 96 well plates (Pall Life Sciences) prior to cytokine measurements. Data were collected using a Bio-Plex Suspension Array Reader (Bio-Rad Laboratories Inc.) and BIO-plex manager software (version 4). HIV-p24 ELISA was performed using thawed macrophage culture supernatants that had been collected and stored at −80 °C, per the manufacture’s instructions (PerkinElmer). GM-CSF ELISA was also performed on culture supernatants per the manufacture’s instructions (Biolegend). Human IL1β levels in the culture supernatants were determined using a Human IL1β Ultrasensitive ELISA kit across three independent donors (Limit of detection: 0.61 pg/mL, Novex Life Technologies).

### Flow cytometry sorting

Mtb-infected macrophages were detached from low attachment plates using pre-warmed trypsin. Cells were pelleted and resuspended in FACS buffer (1× PBS, 2% FBS) and sorted on an Aria IIu. Three populations of macrophages were sorted. From an uninfected culture of macrophages we sorted macrophages as unexposed, uninfected. From the infected macrophages, two populations were sorted corresponding to RFP + GFP + and RFP + GFP- representing macrophages with transcriptionally active Mtb, and macrophages with transcriptionally silent Mtb. Cells were sorted directly into RLT buffer (Qiagen) prior to filtration for preparation for RNA sequencing. This experiment was conducted with three independent donors on three separate occasions.

### Flow cytometry analysis

Differentiated macrophages were detached from low attachment plates using pre-warmed trypsin. Cells were pelleted and resuspended in FACS buffer (1× PBS, 2% FBS) and blocked with TruStain FcX (Biolegend, Cat: 422301, 5 uL per 1 million cells) for 10 min at room temperature. The cells were subsequently stained with:

Panel 1: CD11b-BV421 (Biolegend, Cat: 101235), CD163-PE (Biolegend, Cat: 326505), CD32-PECy7 (Biolegend, Cat: 303213), CD36-APC (Biolegend, Cat: 336207), HLA-DR-BV510 (Biolegend, Cat: 307645)

Panel 2: CD11b-BV421 (Biolegend, Cat: 101235), CD274-PE (Biolegend, Cat: 329705), CD206-PECy7 (Biolegend, Cat: 321123), CD86-APC (Biolegend, Cat: 305405), CD64-BV510 (Biolegend, Cat: 305057) and analyzed on an LSR II. All antibodies were used at a 1:20 dilution.

### RNA sequencing

Infected macrophages were isolated as described, and RNA was purified using the RNeasy kit according to the manufacturer’s instructions (Qiagen). RNA was quantified by Nanodrop and quality assessed by Agilent 2100 Bioanalyzer. Libraries were created from high quality RNA using TruSeq RNA Library Prep Kit v2 (Illumina). Libraries were quantified by Qubit and sequenced on a HiSeq 2500 Sequencing System (Illumina). Each sample was sequenced with at least 30 million reads using single-end 50 base pair reads.

### Differential expression analysis

Sequenced reads were aligned to the human reference genome (build hg19 UCSC) using TopHat and Bowtie2. Raw counts were calculated with HTseq using the hg19 Ensembl annotation. Normalization and differential expression analysis was performed using the DESeq2 package for R.

### Functional analysis

Functional analysis of differentially expressed genes was performed using Ingenuity Pathway Analysis (Qiagen). Genes whose average expression changed by two-fold or more (up or down) in the three donors were analyzed using the Causal Networks tool to identify enriched pathways.

### Nitrite production

Culture supernatant was collected from uninfected, Mtb-infected, and GM-CSF stimulated Mtb-infected cells and filtered twice, to remove Mtb, by centrifugation using 0.2um Bio-Inert AcroPrep 96 well plates (Pall Life Sciences) prior to nitrite quantification using a colorimetric nitrate/nitrite assay kit (Cayman Chemicals).

### Quantitative PCR

Infected macrophages (untreated, GM-CSF treated, or Vitamin D treated) were lysed in Trizol for trizol-chloroform preparation of RNA. RNA was precipitated using isopropanol and then processed using Qiagen RNEasy columns (Qiagen). RNA was reverse transcribed into cDNA using SuperScript III (ThermoFisher Scientific) and quantitative PCR for hCAMP and GAPDH was done using iTAQ Universal Sybr Green (Bio-Rad). The primer sequences for GAPDH are: F: 5’-GTCTCCTCTGACTTCAACAGCG-3’ and R: 5’-ACCACCCTGTTGCTGTAGCCAA-3’. The primer sequences for hCAMP are: F: 5’-GGACCCAGACACGCCAAA-3’, R: 5’-GCACACTGTCTCCTTCACTGTGA-3’.

## Supplementary information

Supplementary Information

Description of Additional Supplementary Files

Supplementary Data 1

Supplementary Data 2

Source Data

## Data Availability

RNA sequencing data that support the results of this study have been deposited in GEO with the primary accession codes [GSE131220]. The source data underlying Figs. 1c, 1d, 1f, 2c, 2d, 2e, 2f, 3h, 3i, 3j, 4a, 4b, 4c, 4d, 4e, and Supplementary Figs 1, 2a, 2b, 2c, 2d, 4a, 4b, 4c, 4d, 4e, 4f, 5, 6, 7, 8a and 8b are provided as a Source Data file. A reporting summary for this Article is available as a Supplementary Information file.
