# Supplementary material for: Heterogeneous GM-CSF signaling in macrophages is associated with control of Mycobacterium tuberculosis
Source: Nat Commun. 2019 May 27;10:2329. doi: 10.1038/s41467-019-10065-8 (PMC6536549; doi:10.1038/s41467-019-10065-8)
Supplement: Supplementary file 1 — Supplementary Information [file 41467_2019_10065_MOESM1_ESM.pdf]

“Heterogeneity in GM-CSF signaling is associated with control of Mycobacterium tuberculosis”

Bryson et al

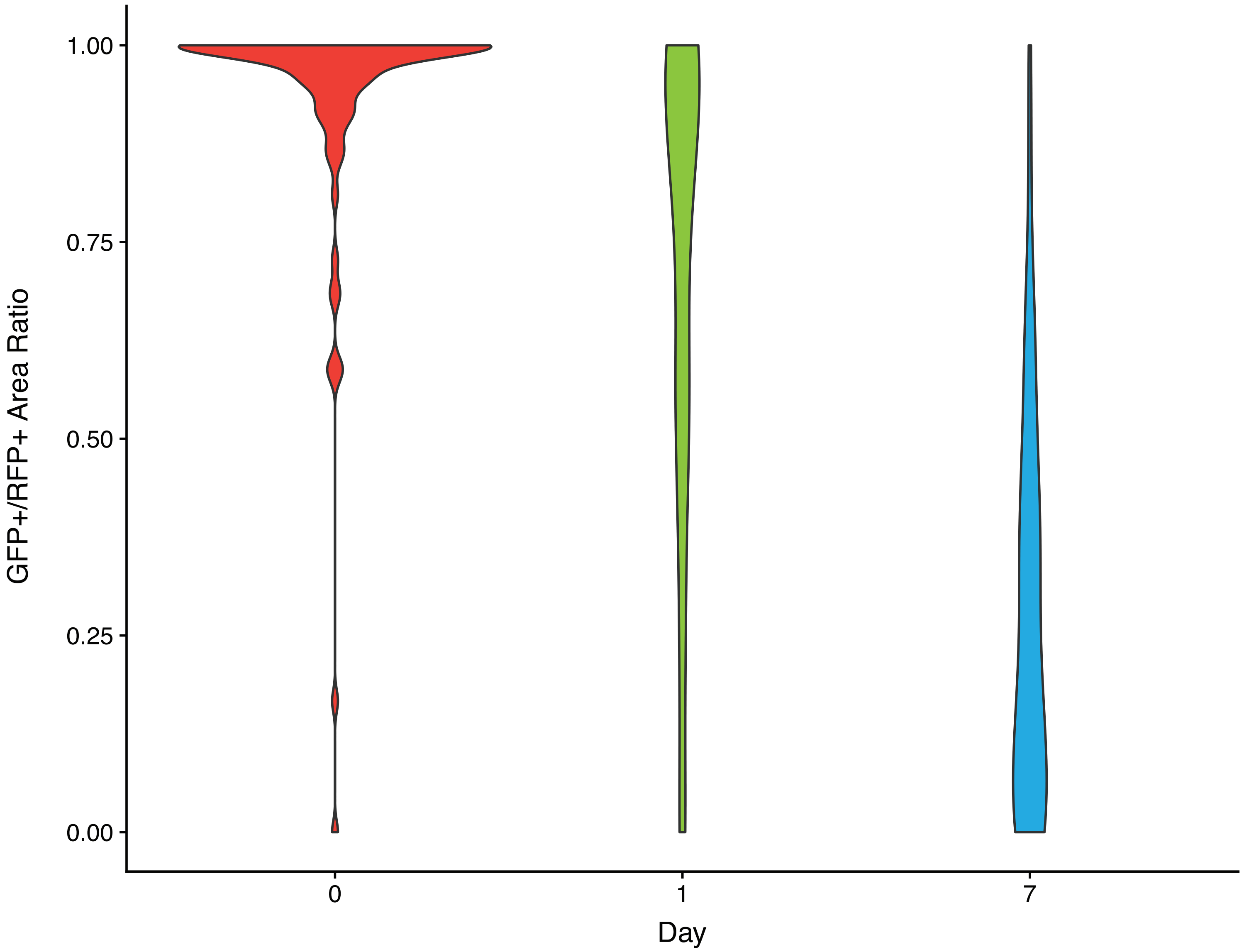

Supplemental Figure 1 Time course analysis of Mtb viability reporter in human monocyte-derived macrophages over time. Macrophages were infected with Mtb expressing the live-dead reporter. aTc was added either immediately after washing away extracellular bacteria (Day 0), one day after infection (Day 1), or seven days after infection (Day 7). Bacterial viability was assessed by quantifying the area of GFP+ pixels relative to the area of RFP+ pixels within an individual macrophage. Source data are provided as a Source Data File.

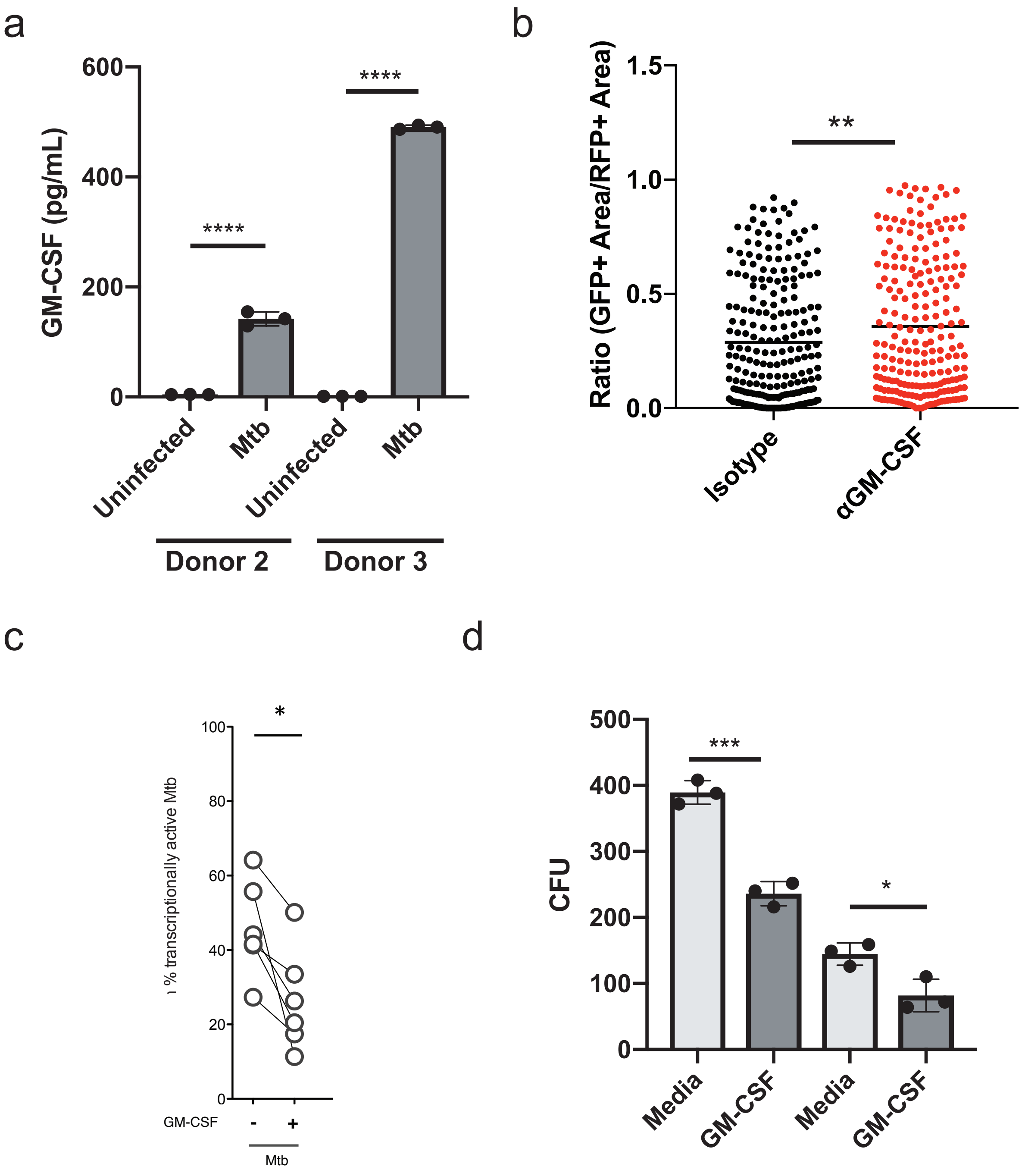

Supplemental Figure 2 GM-CSF revealed as critical regulator of macrophage control of Mtb. (a) Extracellular GM-CSF was measured and compared between paired Mtb-only and uninfected cultures at 24 hours post-Mtb infection. Comparisons are two-tailed paired t-test. (b) Mtb-infected macrophages were scored 4 days post-infection based on the percentage of transcriptional activity following pre-treatment with anti-GM-CSF antibody or isotype control. (c) Mtb-infected macrophages were scored 4 days post-infection based on the percentage of transcriptional activity of the untreated or GM-CSF treated macrophages. Circle represents the median. Comparisons made using the Mann-Whitney test. (d) Measurements of bacterial survival after GM-CSF treatment using colony-forming units. Comparisons are two-tailed paired t-test. (*, $p<0.05$; **, $p<0.01$, ***, $p<0.001$, ****, $p<0.0001$). Source data are provided as a Source Data File.

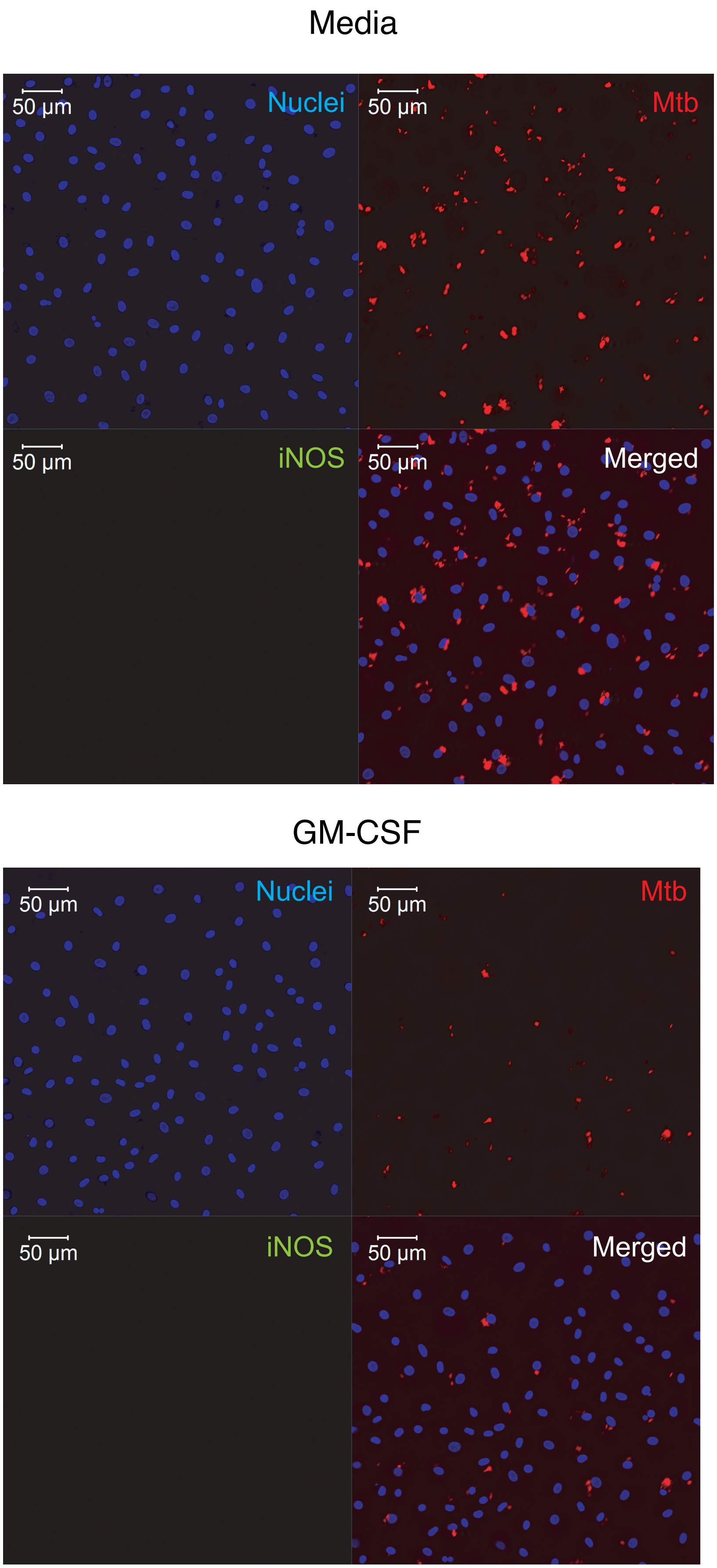

Supplemental Figure 3 Immunofluorescence staining for iNOS in human monocyte-derived macrophages. Mtb-infected macrophages were treated with GM-CSF and subsequently fixed for detection of iNOS by immunofluorescence staining.

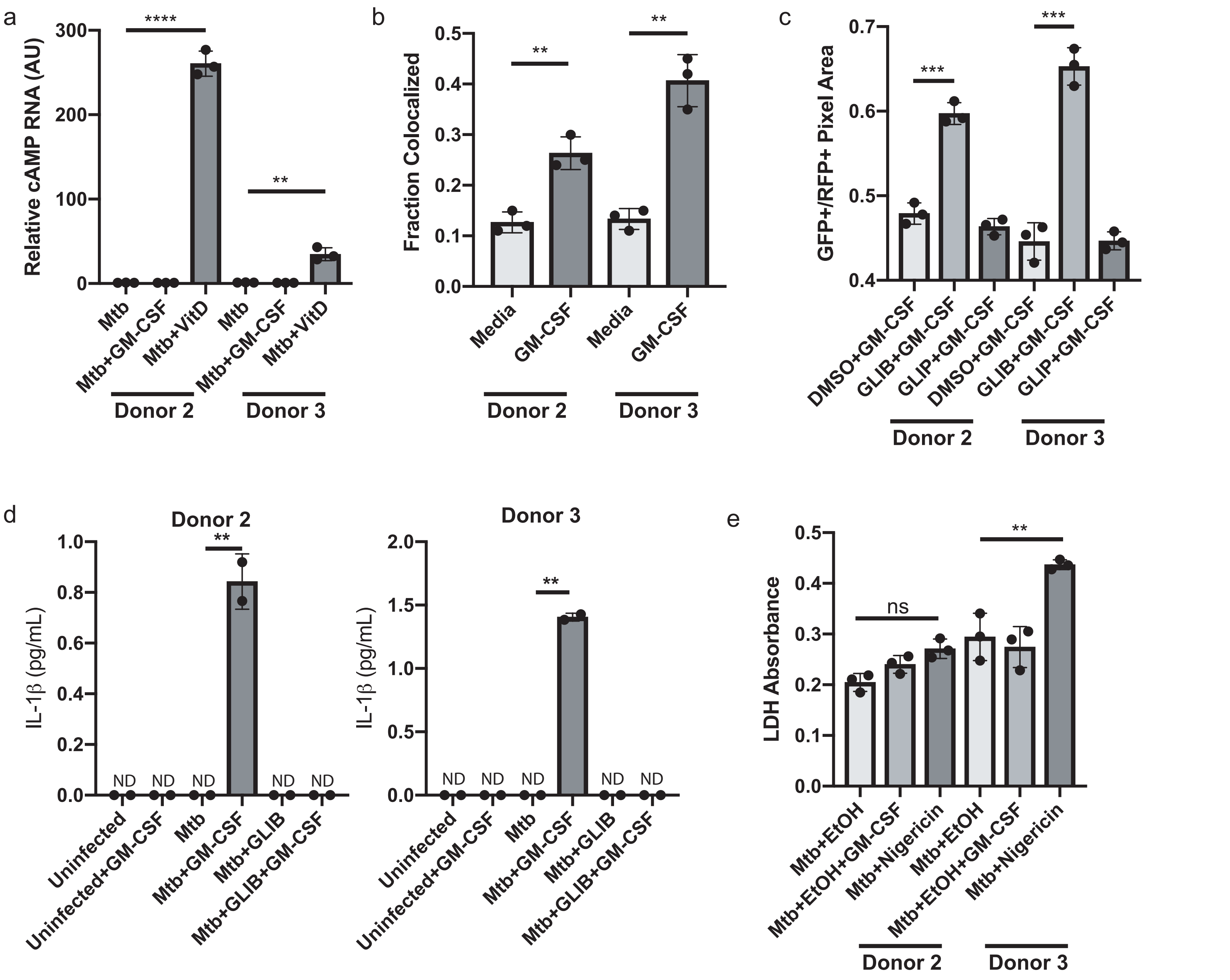

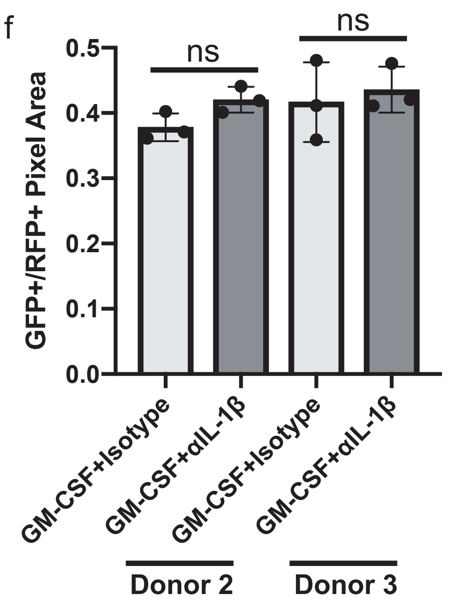

Supplemental Figure 4 GM-CSF stimulates phagolysosomal fusion and IL-1β secretion in the absence of antimicrobial peptide production or NO production. (a) Quantitative PCR (qPCR) analysis of CAMP mRNA expression in primary human macrophages were treated with GM-CSF or active Vitamin D, a known modulator of antimicrobial peptide expression. Results are presented in-terms of a fold change after normalizing with GAPDH mRNA. Each value represents the mean of ± standard deviation of three independent experiments. (b) Phagolysosomal colocalization of GFP-Mtb with Lysotracker red puncta was measured in 200–600 macrophages per well. (c) The effect of glibenclamide or glipizide on GM-CSF mediated control was compared between vehicle-treated, glipizide-treated or glibenclamide treated cultures. (d) Extracellular IL-1β production was measured and compared between paired unstimulated and GM-CSF stimulated Mtb-infected macrophages with respective inhibitors. Error bars represent standard deviation of the technical replicates. (e) LDH absorbance values as a metric of pyroptosis induction in unstimulated Mtb-infected macrophages, GM-CSF treated Mtb-infected macrophages, and nigericin treated Mtb-infected macrophages. Error bars represent standard deviation of the technical replicates. (f) Quantification of bacterial survival (GFP+ area/RFP+ area) in the setting of GM-CSF with isotype antibody or IL-1β antibody. Error bars represent standard deviation of the technical replicates. (ns, not significant, *, $p<0.05$, **, $p<0.01$, ***, $p<0.001$, ****, $p<0.0001$). Source data are provided as a Source Data File.

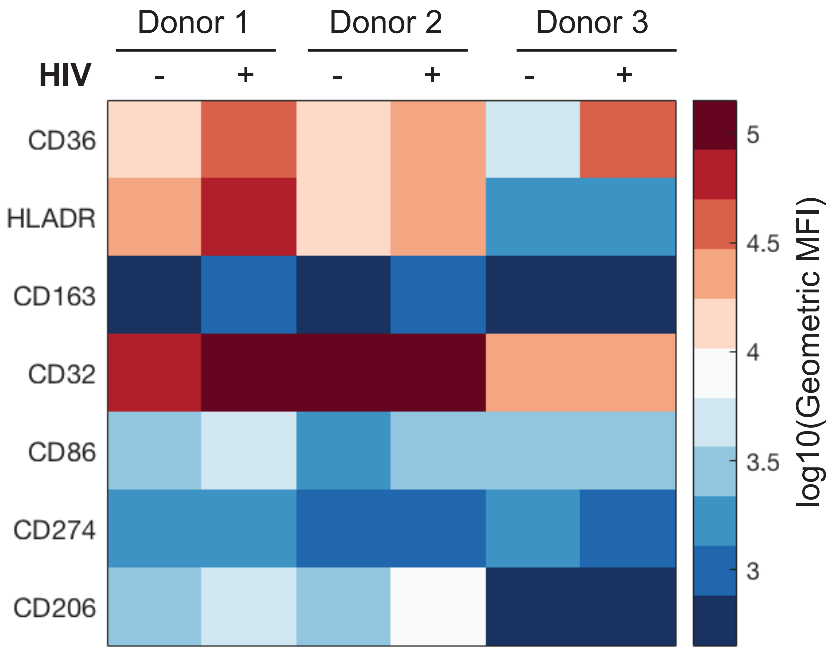

Supplemental Figure 5 HIV coinfection strongly alters surface expression of CD32 and CD36. Surface expression analysis of CD163, CD32, CD36, HLA-DR, CD274, CD206, CD86 on human serum-derived macrophages with or without HIV infection. Source data are provided as a Source Data File.

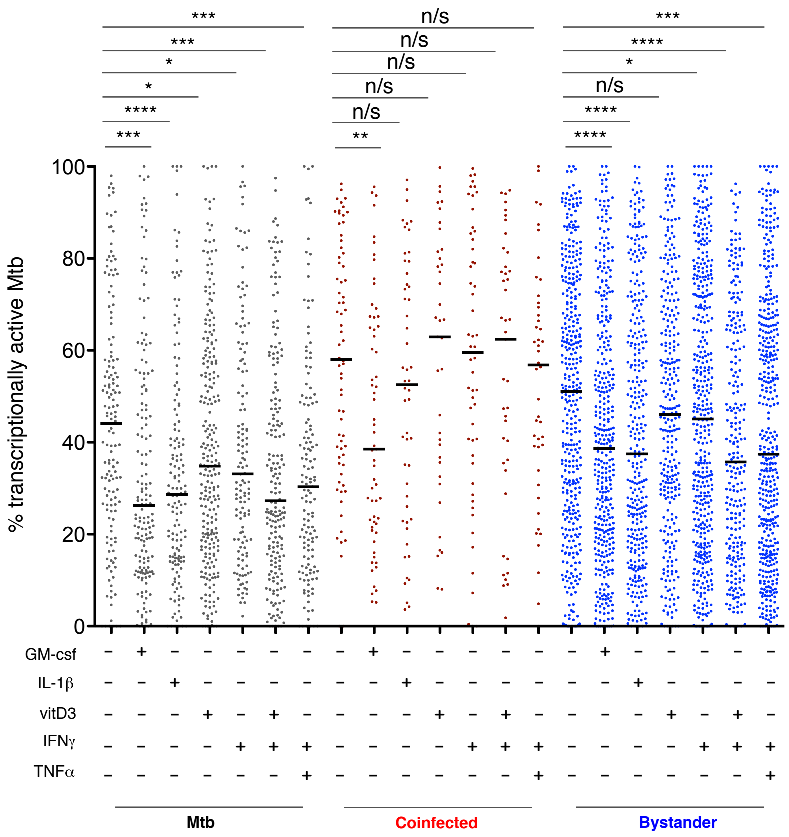

Supplemental Figure 6 GM-CSF enhances control of Mtb in HIV co-infected macrophages. Donor 1. Mtb-infected macrophages were scored 4 days post-infection based on the percentage of transcriptional activity of the resident Mtb and compared to paired co-infected and bystander cells, with or without treatment with various effectors. Black lines represent the median. Dots represent data from an individual macrophage. Comparisons made using the Mann-Whitney test. (*, $p<0.05$, ** $p<0.01$, *** $p<0.001$, **** $p<0.0001$, n/s, not significant). Source data are provided as a Source Data File.

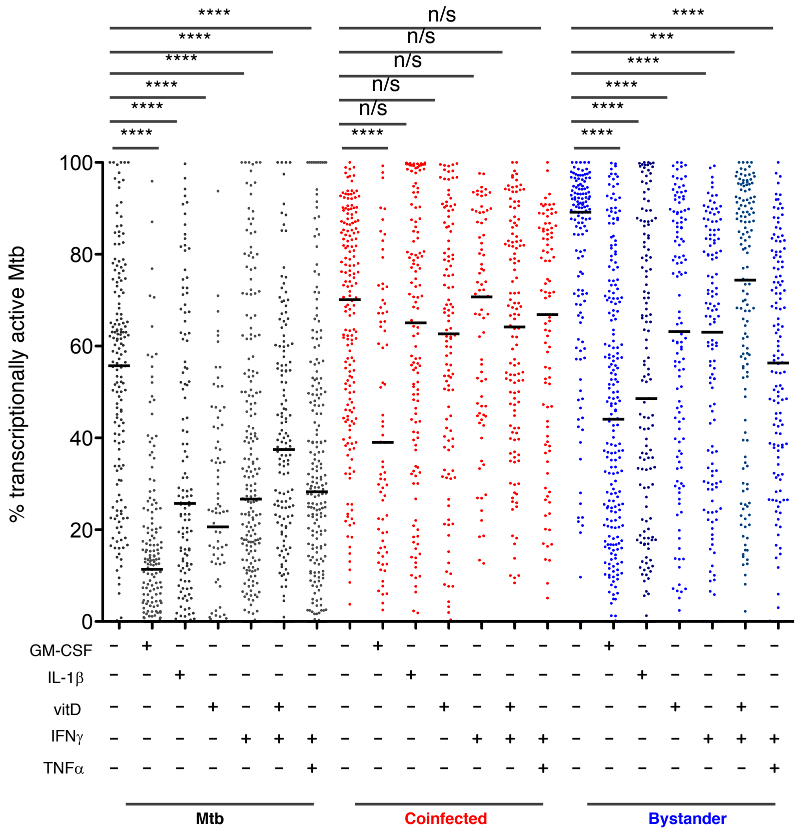

Supplemental Figure 7 GM-CSF enhances control of Mtb in HIV co-infected macrophages. Donor 2. Mtb-infected macrophages were scored 4 days post-infection based on the percentage of transcriptional activity of the resident Mtb and compared to paired co-infected and bystander cells, with or without treatment with various effectors. Black lines represent the median. Dots represent data from an individual macrophage. Comparisons made using the Mann-Whitney test. (*, p<0.05, ** p<0.01, *** p<0.001, **** p<0.0001, n/s, not significant). Source data are provided as a Source Data File.

a

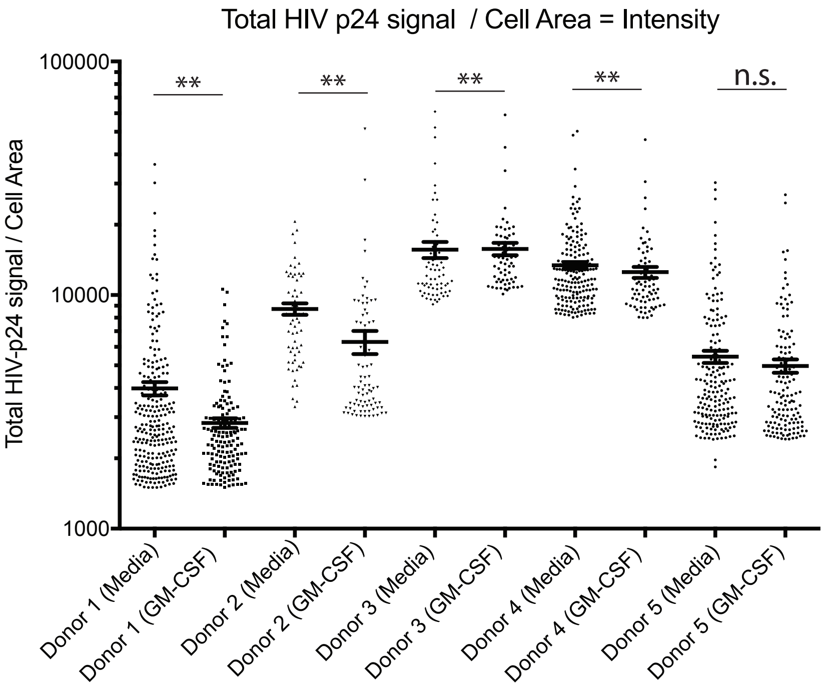

b

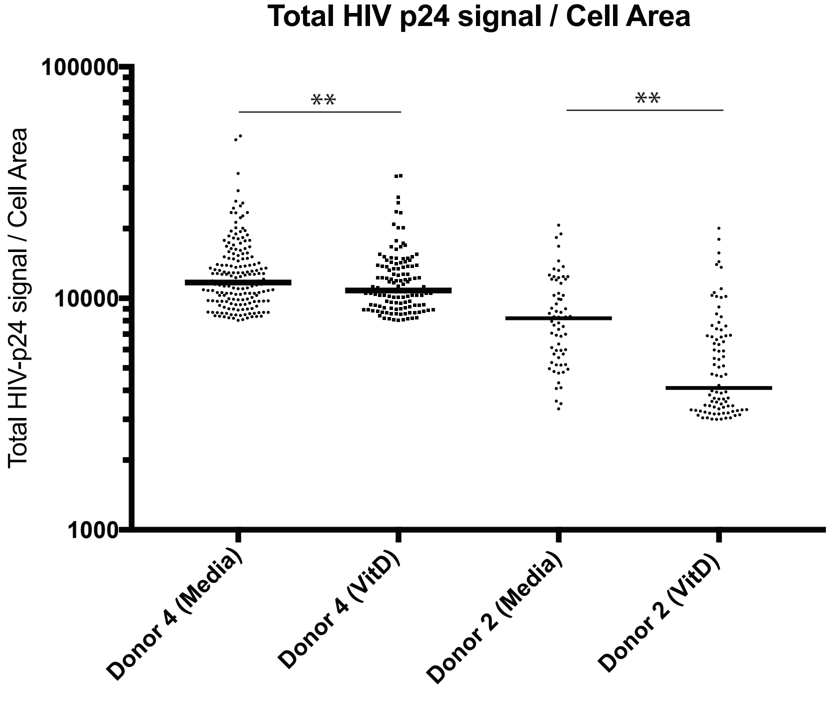

Supplemental Figure 8 GM-CSF and active Vitamin D reduce p24 staining in human monocyte-derived macrophages (a) Quantification of p24 signal in macrophages with or without GM-CSF treatment. (b) Quantification of p24 signal in macrophages with or without active vitamin D treatment. Comparisons made using the Mann-Whitney test. (** p<0.01, *** n.s, not significant). Source data are provided as a Source Data File.
