# Supplementary material for: Heterogeneous GM-CSF signaling in macrophages is associated with control of Mycobacterium tuberculosis
Source: Nat Commun. 2019 May 27;10:2329. doi: 10.1038/s41467-019-10065-8 (PMC6536549; doi:10.1038/s41467-019-10065-8)
Supplement: Supplementary file 2 — Description of Additional Supplementary Files [file 41467_2019_10065_MOESM2_ESM.docx]

**Description of Supplementary Files**

**File Name:** Supplementary Data 1

**Description:** RNA sequencing summary. DESeq2 results comparing different macrophage subpopulations (RFP-/GFP- vs RFP+/GFP+, RFP-/GFP- vs RFP+/GFP-, RFP+/GFP+ vs RFP+/GFP-).

**File Name:** Supplementary Data 2

**Description:** Results of Ingenuity Pathway Analysis Causal Network analysis comparing pathways differentially activated between macrophage subpopulations.
